# Current status of gonococcal antimicrobial susceptibility with special reference to Azithromycin and Ceftriaxone: Report from a tertiary care hospital in Bangladesh

**DOI:** 10.12669/pjms.346.16144

**Published:** 2018

**Authors:** Mahmuda Naznin, Md. Abdus Salam, Md. Zahid Hossain, Md. Shah Alam

**Affiliations:** 1 *Mahmuda Naznin, MPhil. Lecturer, Department of Microbiology, Rajshahi Medical College, Rajshahi-6000, Bangladesh*; 2 *Md. Abdus Salam, PhD, FRCP. Professor of Microbiology, Shaheed Ziaur Rahman Medical College, Bogura, Bangladesh*; 3 *Md. Zahid Hossain, MBBS. Lecturer, Department of Virology, Rajshahi Medical College, Rajshahi-6000, Bangladesh*; 4 *Md. Shah Alam, MPhil. Professor, Department of Microbiology, Rajshahi Medical College, Rajshahi-6000, Bangladesh*

**Keywords:** Gonorrhea, Antimicrobial resistance, Azithromycin, Ceftriaxone, Tertiary care hospital, Bangladesh

## Abstract

**Objectives:**

Successful treatment of gonorrhea has always been jeopardized by the emergence of resistance to antibiotics recommended as first-line therapies. The present investigation was carried out to demonstrate the current status of antimicrobial susceptibility of *N. gonorrhoeae* with a special reference to azithromycin and ceftriaxone.

**Methods:**

Microscopical detection in Gram-stained smear and isolation by culture in Thayer-Martin medium were done for 60 clinically suspected gonorrhea patients using urethral discharge or prostatic secretion for male and endocervical secretion for female. Isolates of *N. gonorrhoeae* were subjected to antimicrobial susceptibility testing by modified Kirby Bauer disk diffusion method against eight antimicrobial drugs including azithromycin and ceftriaxone.

**Results:**

Culture yielded a total of 25(42%) isolates of *N. gonorrhoeae* from 60 clinically suspected patients of both sexes; 21 from male (17 from urethral discharge and 04 from prostatic secretion) and 04 from female (endocervical secretion). Isolates of *N. gonorrhoeae* showed moderate to high resistance (60 to 88%) to penicillin, tetracycline, cotrimoxazole, erythromycin, ciprofloxacin and cefixime. While resistance to azithromycin and ceftriaxone was 60% and 48% respectively, which was also moderate.

**Conclusion:**

Our findings indicate moderate to the high resistance of *N. gonorrhoeae* to conventional antibiotics. It also showed moderate resistance to azithromycin and ceftriaxone, current dual therapy recommended by the WHO for the treatment of genital gonorrhea, which is alarming.

## INTRODUCTION

Gonorrhea caused by *Neisseria gonorrhoeae* (*N. gonorrhoeae*) is the second most common sexually transmitted infection (STI) worldwide. The precise global burden of *N. gonorrhoeae* is difficult to establish because of the lack of diagnostic facility and/or reporting systems in many parts of the world. The World Health Organization (WHO) estimates that in each year 78 million new cases of gonorrhea occur among adolescents and adults aged 15-49 years worldwide with a global incidence rate of 19 per 1000 females and 24 per 1000 males. In South-East Asia, each year 11.4 million new cases of gonorrhea were estimated.^1^ In Bangladesh, the prevalence rate of gonorrhea is not well documented but it was found to be 27.81% among STIs and second only to non-gonococcal urethritis (NGU) noted in a retrospective large-scale study incorporating data from 2003 to 2011 at a tertiary care hospital.^2^ Gonorrhea was found to be much higher (35.8%) among female sex workers in Bangladesh.^3^

Uncomplicated gonococcal infection commonly manifests as genital tract infections in both male and female but if untreated can lead to serious complication like infertility in both gender.^4^ The risk of complication increases with repeated gonococcal infection and it also increases the risk for HIV acquisition and transmission.^5^ Gram-stained smears can provide a presumptive diagnosis of gonorrhea, however, culture of urethral discharge and/or prostatic secretion in case of male and endocervical swab in case of female is the gold standard for the detection of *N. gonorrhoeae* with high sensitivity and specificity.^6^

*N. gonorrhoeae* is a very well-known pathogen for its ever-increasing antimicrobial resistance, and more resistance is being observed especially in areas where ineffective treatment regimens are practiced. Control of gonorrhea largely relies on detection of cases followed by therapy however, successful treatment has always been hampered by emergence of resistance to each of the antibiotics recommended as first-line therapies. The WHO Global Gonococcal Antimicrobial Surveillance Programme (WHO GASP), monitors trends in drug-resistant gonorrhea and its data from 2009 to 2014 find widespread resistance to ciprofloxacin, increasing resistance to azithromycin (81%), and the emergence of resistance to the current last-resort treatment; the extended-spectrum cephalosporins (66%), oral cefixime or injectable Ceftriaxone.^7^ To minimize the development and spread of resistance, dual therapy consisting of ceftriaxone and azithromycin has been recommended recently as the first-line therapy in international guidelines. However, the first treatment failure with dual therapy was reported in 2016 in the United Kingdom and soon thereafter, a few more countries from Europe, America and Australia also reported various degree of resistance to dual therapy.^8^,^9^ To explore the current status of antimicrobial resistance of *N. gonorrhoeae* with special reference to azithromycin and ceftriaxone in Bangladesh, we conducted this cross-sectional investigation at a tertiary care teaching hospital.

## METHODS

### Patients

This was a descriptive cross-sectional investigation of clinically suspected patients of gonorrhea. The protocol of this investigation was approved by the ‘Ethical Review Committee’ of Rajshahi Medical College, Bangladesh for ethical issues related to this research. Purposive sampling technique was applied to include 60 clinically suspected cases of gonorrhea in male (15-55 yrs.) and female (15-45 yrs.) attending at the outpatient departments of Dermatology & Venereology and Gynecology of Rajshahi Medical College Hospital from January to December 2016. Relevant clinical and socio-demographic data were collected through clinical examination and personal interview and recorded systematically. Laboratory tests were done at the Department of Microbiology, Rajshahi Medical College, Bangladesh.

### Samples and Culture

After obtaining informed written consent and taking all aseptic precautions, urethral swabs (60), prostatic secretion swabs (40) and endocervical swabs (20) were collected in duplicate from 60 clinically suspected cases at the department of Microbiology following standard techniques of collection. First collected swab in each case was inoculated onto the Thayer-Martin medium (Oxoid, UK) aseptically following multiple strokes technique soon after collection and was incubated at 37°C in 5-10% CO_2_ for 24 to 48 hours. CO_2_ was provided by a candle jar. Isolates of *N*. *gonorrhoeae* were identified based on colonial morphology (growth of typical colonies), Gram stain (Gram-negative intracellular diplococci in stained smears), and oxidase reaction (oxidase-positive). Sugar fermentation test was further performed to confirm *N*. *gonorrhoeae* as glucose fermenter. The second swab samples were utilized for smear preparation in Gram staining to see Gram-negative intra or extracellular diplococci under bright field microscope.^10^

### Antimicrobial Susceptibility Testing

Antimicrobial susceptibility testing (AST) was performed onto Chocolate agar medium by modified Kirby Bauer disk diffusion method with inoculums of 0.5 McFarland standards.^10^ Antimicrobial disks (Oxoid, UK) of penicillin(10µg), tetracycline (30µg), erythromycin(15µg), cotrimoxazole(25µg),ciprofloxacin(5µg), cefixime (5µg), azithromycin (15µg) and ceftriaxone(30µg) were selected as per Clinical and Laboratory Standard Institute (CLSI) 2016 guideline.^11^ Inoculated plates with the antimicrobial disks were incubated at 37°C for 18-24 hours in a humid atmosphere with 5-10% CO_2_ provided by a candle jar. The diameters of the zones of inhibition around disks were measured in millimeter. Inhibition zone produced by each disk was considered into two susceptibility categories namely Sensitive (S) and Resistant (R) according to CLSI guideline.^11^ *Staphylococcus aureus* ATCC-25923 was used for quality control in the interpretation of zone of inhibition by antimicrobial disk.

### Statistical Analysis

All data were entered into Statistical Package for Social Sciences (SPSS) version 21.0. Mean ± standard deviation (SD) was calculated for age in both genders. Frequencies with percentage were generated for categorical variables such as, number positive in laboratory tests, sociodemographic profiles of patients, number of antimicrobial-resistant cases etc.

## RESULTS

Laboratory findings and their correlation with sociodemographic profile of gonorrhea patients are shown in Table-I. The rate of microscopic detection and isolation in culture of *N*. *gonorrhoeae* was 36(60%) and 25(42%) respectively. Both microscopy and culture yielded higher rate of detection from urethral discharge followed by endocervical and prostatic secretions. There was male preponderance (21 vs. 04) among culture-positive gonorrhea with highest detection rate found among 15-24 years age group in both sexes. Mean ± SD age in years for male and female was 27.52±70.55 and 23.50±4.90 respectively. Occupation wise highest number 09(43%) of gonorrhea cases was from student in case of male and housewife 03(75%) in case of female among seven different categories of occupations. Overwhelming majority (60%) of cases were from lower class (monthly income in BDT. 10000-15000) followed by middle class (40%) (monthly income in BDT. 15001-20000) for their socioeconomic status.

**Table-I:** Laboratory findings and their correlation with sociodemographic profile of gonorrhea patients (n=25)

Parameters	Number (%)
***No. of samples investigated***	60 (100)
Urethral discharge	30 (50)
Prostatic secretion	20 (33)
Endocervical secretion	10 (17)
***No. of positive cases by microscopy***	36 (60)
Positive urethral discharge	25(70)
Positive prostatic secretion	07(19)
Positive endocervical secretion	04 (11)
***No. of positive cases by culture***	25 (42)
Positive urethral discharge	17 (68)
Positive prostatic secretion	04 (16)
Positive endocervical secretion	04 (16)
***Male***	50 (83)
Age range (Yrs.)	15-55
Mean±SD	27.52±70.55
No. culture positive in 15-24 age group	10 (48)
No. culture positive in 25-34 age group	06 (29)
No. culture positive in 35-44 age group	04 (19)
No. culture positive in 45-55 age group	01 (04)
***Female***	10 (17)
Age range (Yrs.)	15-34
Mean±SD	23.50±4.90
No. culture positive in 15-24 age group	03 (75)
No. culture positive in 25-34 age group	01 (25)
***Socioeconomic class***	
Lower class	15 (60)
Middle class	10 (40)
***Occupation***	
Student	09 (36)
Service	05 (20)
Farmer	04 (16)
Housewife	03 (12)
Day laborer	02 (08)
Small trader	01 (04)
Driver	01 (04)

Antimicrobial susceptibility testing results of *N*. *gonorrhoeae* isolates are shown in Table-II. It is evident from the results that *N. gonorrhoeae* showed moderate to high resistance (60 to 88%) to conventional antibiotics; penicillin (88%), tetracycline (80%), cotrimoxazole (80%), erythromycin (72%), ciprofloxacin (68%) and cefixime (60%). While resistance to azithromycin and ceftriaxone was 60% and 48% respectively, which was also considered as moderate resistance.

**Table-II:** Antimicrobial susceptibility testing of *N. gonorrhoeae* isolates (n =25).

Antimicrobials	Sensitive No. (%)	Resistant No. (%)
Penicillin (10 µg)	03(12)	22(88)
Tetracycline (30 µg)	05(20)	20(80)
Cotrimoxazole (25 µg)	05(20)	20(80)
Erythromycin (15 µg)	07(28)	18(72)
Ciprofloxacin (05 µg)	08(32)	17(68)
Cefixime (05 µg)	10(40)	15(60)
Azithromycin (15 µg)	10(40)	15(60)
Ceftriaxone (30 µg)	13(52)	12(48)

## DISCUSSION

Gonorrhea is a sexually transmitted infection affecting comparatively younger age group and 15-24 years age group constituted the highest number in both gender in the present investigation. Further, people from low income group were more vulnerable to contract gonorrhea. These sociodemographic factors contributing to higher prevalence rate of gonorrhea were similar as documented in different reports.^12^,^13^ The reasons of common sociodemographic factors worldwide may be well correlated with the facts that people of this age and social class groups are more sexually active, mostly unmarried and unemployed and also largely influenced by widespread and easy availability of internet-accessible electronic gadgets. All these factors are strong attributes for their unprotected and promiscuous sexual activities to contract gonococcal infection.

Regarding antimicrobial susceptibility, we observed moderate to high (60-88%) resistance to conventional antimicrobials including ciprofloxacin and cefixime and similar trend of resistance has been reported over the time by many investigators.^14^,^15^,^16^ *N. gonorrhoeae* is a well-adapted human pathogen and notorious for its ever changing antimicrobial susceptibility for decades. Penicillin, sulphonamides and tetracycline are of no use in the treatment of gonorrhea since long. In 1993, ciprofloxacin, a fluoroquinolone, and two cephalosporins (ceftriaxone and cefixime) were the recommended treatments for gonorrhea. However, in the late 1990s and early 2000s, ciprofloxacin resistance was detected and within a few years it became widespread in different countries that warned US Centre for Disease Control (CDC)in 2007 to stop its recommendation as empiric treatment for gonorrhea.^17^ Again following continued declines in cefixime susceptibility, CDC updated its recommendations in 2012 to recommend injectable ceftriaxone plus either azithromycin or doxycycline as the only first-line treatment.^18^ In 2015, CDC’s STD treatment guidelines recommended only one regimen of dual therapy for the treatment of gonorrhea; the injectable cephalosporin, ceftriaxone, plus oral azithromycin.^7^ Unfortunately, as it always goes with *N*. *gonorrhoeae*, this latest regimen also turns to be ineffective at least partially with the prevalence of *N*. *gonorrhoeae* found resistant to azithromycin (RTA) and decreased susceptibility to ceftriaxone (DSC) detected in China in a large scale retrospective study and also reported from a few countries in Europeand the Americas.^9^,^12^,^19^,^20^ Similar in the line, we also noted moderate resistance to azithromycin (60%) and ceftriaxone (48%), the current dual therapy for gonorrhea in our present investigation. Our results show much higher rate of resistance for these two drugs in comparing with contemporary research findings which is alarming though but not unusual for a pathogen like *N. gonorrhoeae*. It is unclear whether circulating gonococcal strains with altered susceptibility to the key antibiotics acquired mutations through domestic selective pressure in Bangladesh or whether the strains have been imported. Experience says that both are possible here because macrolides like azithromycin has been used very frequently for trivial causes and it is also the antibiotic of choice for a number of other common bacterial infections. Similarly, the ceftriaxone is also being used as first line antibiotic for many bacterial and non-bacterial etiologies in both community and hospitals unfortunately. Moreover, all antibiotics including these two very potential drugs are always available as over the counter drugs in Bangladesh. As a result it is quite logical to consider the strong probability of antibiotic abuse leading to drug resistance acquired by *N. gonorrhoeae* through selective pressure. Another very alarming and unethical practice of antibiotic abuse in Bangladesh is its unrestricted use in poultry and cattle farming which is thought to be an important attribute for developing increasing antibiotic resistance in human treatment. Besides, because of increasing globalization and migration of people now-a-days, increased resistance to antibiotics due to imported drug-resistant gonococcal strains can’t also be ruled out.

Increased resistance to most antibiotics has raised concerns about the eventual development of untreatable gonococcal infections with serious sexual and reproductive health consequences. To address the changing situation, gonococcal treatment guidelines need to be updated. It is strongly recommended that countries like Bangladesh should establish standard national treatment protocols based on the local epidemiological and antimicrobial susceptibility data. Further, immediate introduction of ban on selling antibiotics as over the counter drugs, practice of strict antibiotic policies, enhancing facilities for proper laboratory diagnosis with antimicrobial susceptibility could be additional measures to stop drug resistance in gonorrhea.

### Limitations

There are some potential limitations of present investigation. First, this was a cross-sectional survey taking very small number of patients attending at a tertiary care facility, so it doesn’t represent the actual situation of country-wide antimicrobial resistance to *N. gonorrhoeae*. Second, we couldn’t determine minimal inhibitory concentration (MIC) of antibiotics especially ceftriaxone, so it might have been an over estimation for resistance to ceftriaxone. Third, there was no scope to carry out molecular typing of *N. gonorrhoeae* isolates. Future studies should include molecular and genomic analyses to investigate gonorrhea transmission and to track the spread of antimicrobial resistance.

## CONCLUSIONS

Current status of gonococcal antimicrobial susceptibility has revealed moderate resistance to azithromycin and ceftriaxone, dual therapy recommended by the WHO for the treatment of genital gonorrhea in the present investigation which is quite alarming. Selection of gonorrhea treatment should ideally be guided by culture and sensitivity test and empirical therapy must be considered based on recent antibiogram of a particular geographical area. Reevaluation of present treatment guideline is urgent before gonorrhea becomes an untreatable condition especially in countries like Bangladesh.
